# Effects of dietary beef, pork, chicken and salmon on intestinal carcinogenesis in A/J Min/+ mice

**DOI:** 10.1371/journal.pone.0176001

**Published:** 2017-04-20

**Authors:** Christina Steppeler, Marianne Sødring, Bjørg Egelandsdal, Bente Kirkhus, Marije Oostindjer, Ole Alvseike, Lars Erik Gangsei, Ellen-Margrethe Hovland, Fabrice Pierre, Jan Erik Paulsen

**Affiliations:** 1 Department of Food Safety and Infection Biology, Norwegian University of Life Sciences, Oslo, Norway; 2 Department of Chemistry, Biotechnology and Food Science, Norwegian University of Life Sciences, Ås, Norway; 3 Nofima, Norwegian Institute of Food, Fisheries and Aquaculture Research, Ås, Norway; 4 Animalia–Norwegian Meat and Poultry Research Centre, Oslo, Norway; 5 INRA UMR1331 Toxalim (Research Center in Food Toxicology), University of Toulouse, Toulouse, France; National Institute for Agronomic Research, FRANCE

## Abstract

The International Agency for Research on Cancer has classified red meat as “probably carcinogenic to humans” (Group 2A). In mechanistic studies exploring the link between intake of red meat and CRC, heme iron, the pigment of red meat, is proposed to play a central role as a catalyzer of luminal lipid peroxidation and cytotoxicity. In the present work, the novel A/J Min/+ mouse was used to investigate the effects of dietary beef, pork, chicken, or salmon (40% muscle food (dry weight) and 60% powder diet) on *Apc*-driven intestinal carcinogenesis, from week 3–13 of age. Muscle food diets did not differentially affect carcinogenesis in the colon (flat ACF and tumors). In the small intestine, salmon intake resulted in a lower tumor size and load than did meat from terrestrial animals (beef, pork or chicken), while no differences were observed between the effects of white meat (chicken) and red meat (pork and beef). Additional results indicated that intestinal carcinogenesis was not related to dietary n-6 polyunsaturated fatty acids, intestinal formation of lipid peroxidation products (thiobarbituric acid reactive substances, TBARS), or cytotoxic effects of fecal water on *Apc*^-/+^ cells. Notably, the amount of heme reaching the colon appeared to be relatively low in this study. The greatest tumor load was induced by the reference diet RM1, underlining the importance of the basic diets in experimental CRC. The present study in A/J Min/+ mice does not support the hypothesis of a role of red meat in intestinal carcinogenesis.

## Introduction

Colorectal cancer (CRC) represents a major global health concern, particularly in developed countries. Besides genetic predispositions, CRC development is influenced by various life style factors and the consumption of red and processed meat has been associated with an increased risk for CRC [1–3]. In 2015, the International Agency for Research on Cancer (IARC) concluded that the evidence on the carcinogenicity of red and processed meat was sufficient to classify processed meat as “carcinogenic to humans” (Group 1) and red meat as “probably carcinogenic to humans” (Group 2A) [4]. In their report, red meat is defined as meat from beef, veal, pork, lamb, mutton, horse, or goat, while processed meat includes all types of meat that have been subjected to flavor enhancement or preservation, e.g. salting, curing, fermentation or smoking. Genotoxicity, lipid peroxidation and the formation of mutagenic N-nitrosamines are discussed as possible molecular mechanisms behind the link between CRC and red and processed meat intake. In this context, heme, the red pigment in red meat, is suggested to play a central role, and function as a catalyzer [5,6]. The Word Cancer Research Fund guidelines advise to limit the intake of red meat to 500 g (corresponding to 700–750 g raw meat) weekly, with very little if any to be processed [7]. Fish consumption, in contrast to red meat consumption, may be inversely related to CRC [7–10], and its protective effect has been attributed to the high contents of n-3 fatty acids and vitamin D [11]. However, the underlying mechanisms are not known in detail, and a few studies have also indicated no connection, or an increased risk of CRC, after intake of marine n-3 fatty acids [1,12]. The effect of poultry consumption on CRC has not been extensively investigated [7], but, if any, protective implications have been suggested [1]. The Word Cancer Research Fund recommendations are to choose poultry and fish instead of red meat [7].

Types and amounts of dietary fat may influence CRC risk, and it has been suggested that n-6 polyunsaturated fatty acids (PUFA) and saturated fat, in contrast to n-3 PUFA, may have unfavorable effects [13]. High dietary fat, in particular saturated fat, has been shown to increase the synthesis of taurine conjugated bile acids that promote the growth of gut bacteria that generate genotoxic H_2_S gas, and increased levels of potentially carcinogenic secondary bile acids [14,15]. Dietary arachidonic acid (C20:4 n-6), or arachidonic acid derived from other n-6 PUFA, is a precursor of mainly pro-inflammatory eicosanoids [13,16], and n-6 PUFA are, just as n-3 PUFA, generally susceptible towards lipid peroxidation [17,18].

The APC multiple intestinal neoplasia (Min/+) mouse has been widely used as a model to study mechanisms of human CRC pathology. On a molecular level, the development of CRC follows a successive multi-step sequence, and is accompanied by an accumulation of genetic and epigenetic alterations [19]. The majority of human sporadic CRC cases (ca. 85%) follow the chromosomal instability pathway, where mutations in one allele of the tumor suppressor gene *APC* (adenomatous polyposis coli) are typically followed by mutations in, or by loss of heterozygosity (LOH) of the remaining allele. This second event defines the rate-limiting step for tumor initiation [20]. Familial adenomatous polyposis (FAP) patients, in contrast, carry a heterozygous germline mutation in the *APC* gene, and only one hit is required for the complete loss of APC. Similarly, Min/+ mice spontaneously develop CRC as a result of an inherited germline truncating mutation in the *Apc* gene (Min allele), followed by the subsequent loss of the wild-type allele [21,22]. Compared to the conventional Min/+ mouse, which is bred on a C57BL/6 genetic background, and mainly develops lesions in the small intestine, the A/J Min/+ mouse, used in the present work, demonstrates increased colonic carcinogenesis and thus, a tumor development more similar to that seen in humans [23].

Based on the hypothesis that red meat has a greater carcinogenic potential than other types of muscle food, the main objective of the present study was to explore the carcinogenic potential of dietary red meat by comparing the effects of beef-, pork-, chicken- and salmon-based diets on the development of intestinal lesions in A/J Min/+ mice. The experimental diets were designed to compare the effects of intact, mildly heated muscle food (40%) mixed into a standardized powder diet (60%). A second objective was to relate dietary fatty acid composition, the amount of fat, and content of heme iron, as well as fecal levels of lipid peroxidation (TBARS), fecal heme and cytotoxicity of fecal water to intestinal carcinogenesis. To our knowledge, the effects of beef, pork, chicken and salmon on spontaneous intestinal carcinogenesis have never been directly compared before.

## Material and methods

### Ethics statement

The experiment was approved by the Norwegian Animal Research Authority (application ID: 7528) and conducted in compliance with local and national regulations on animal experimentation. At the end of the experiment, animals were sacrificed by cervical dislocation and following events were defined as humane endpoints: blood in feces and a weight increase or decrease of more than 15% compared to other animals of the same age. Daily observations guaranteed the detection of unforeseen events.

### Animals

A/J Min/+ mice were maintained in open-top plastic cages under standard laboratory conditions with free access to food and water. Animals were bred in breeding trios (two female A/J wild-type mice and one male A/J Min/+ mouse), and the resulting offspring (male and female) were genotyped and randomly assigned to the experimental diets at the age of 19–21 days. For genotype determination, DNA was extracted from ear punch samples and the following primer set was used for DNA amplification via PCR: MAPC MT (5’-TGAGAAAGACAGAAGTTA -3’), MAPC 15 (5’-TTCCACTTTGGCATAAGGC-3’), and MAPC 9 (5’-GCCATCCCTT- CACGTTAG-3’). Min/+ mice were identified by the 327 bp Min fragment of *Apc*, which is generated in addition to the 618 bp wild-type PCR product [24]. During the experimental period, four to five mice were housed in one cage.

### Study design

To compare the effects of dietary beef, pork, chicken and salmon on intestinal carcinogenesis, A/J Min/+ mice of both sexes (n = 18–19 per group) were fed four main experimental diets (Table 1 and S1 Table). Beef, Pork, Chicken and Salmon contained approximately 40% (dry weight) muscle food and 60% purified powder diet (SDS special diet services, Witham, UK); the latter representing some features of a ‘western style diet’, e.g. low levels of calcium (0.08%), vitamin D (<12 IU/kg) and fiber (1.96%), but was fat-free (S2 and S3 Tables). The fat content of these four diets (15–17% of dry weight) was adjusted to equal the physiological high fat level of Salmon. Accordingly, the effects of the four muscle foods could be compared directly. Since the high fat level of the Chicken diet poorly mimics a chicken-breast based meal, an additional low-fat chicken breast diet (Chicken Low Fat) was included in the study. In previous studies, where heme iron was found to induce intestinal carcinogenesis, ca. 5% or more of the dietary fat was provided in form of n-6 PUFA [6,25]. To gain additional information about the potential role of n-6 unsaturated fat, a Beef n-6 diet was designed. This diet resembled the Beef diet, i.e. it had the same total fat content, but contained 5% safflower seed oil, which is high in n-6 PUFA. An additional group of mice, fed the standard rodent maintenance diet RM1 (Table 1, S1 and S2 Tables), was included as a reference for the mouse model. RM1 was also used, when the A/J Min/+ mouse was described as a relevant model for initiation, promotion and progression of CRC [26]. The experimental period lasted for 10 weeks, from weaning of the A/J Min/+ mice at the age of 19–21 days, until termination at 13 weeks of age. Every day, diet leftovers from the previous day were removed from the cage before fresh food, thawed in the fridge overnight, was provided. Both the initial, halfway and terminal body weights were recorded. During the last week of the experiment, fresh feces was collected. Energy intake during the final week was calculated as average food intake, registered on 5 different days.

**Table 1:** Composition of study diets (dry weights).

	Powder-based muscle food diets						Reference diet
	Salmon	ChickenLow Fat	Chicken	Pork	Beef	Beef n-6	RM1^e^
**Muscle food including fat (% of dry weight)**	40 (salmon, fat not adjusted)	40 (chicken breast, fat not adjusted)	40 (chicken leg, adjusted with chicken fat)	40 (pork, adjusted with pork fat)	40 (beef, adjusted with beef fat)	35 (beef, adjusted with beef fat)	-
**Safflower seed oil**	-	-	-	-	-	5	-
**Powder diet (% of dry weight)**	60	60	60	60	60	60	-
**Energy** **(MJ/kg diet)**^**a**^	22.4	20.8	22.9	22.9	21.9	22.7	14.7
**Fat (g/100g)**^**a**^	15.6	4.5	16.8	16.6	15.6	13.6	2.7
** SFA**^**b**^	2.2	1.4	4.8	5.8	7.8	5.3	0.5
** MUFA**^**b**^	8.1	2.0	7.5	7.8	6.2	4.6	0.9
** PUFA**	4.8	0.9	4.1	2.7	0.5	2.8	0.8
** n-6/n-3**	1.1	8.6	9.2	7.5	2.9	28.6	11.5
**Protein (g/100 g)**^**a**^	36.2	47.5	37.2	39.4	38.8	39.2	14.4
**sugar as glucose after hydrolysis (g/100 g)**^**a**^	28.7	25.8	24.6	24.2	25.0	25.2	49.0
**Fiber (g/100 g)**^**a**^	2.2	1.5	1.8	1.4	1.7	1.9	17.1
**Calcium (g/100 g)**^**c**^	0.06	0.06	0.06	0.07	0.07	0.06	0.73
**Vit D3 (µg/100 g)**^**a**^	6.0	0.6	0.8	0.5	0.6	0.5	15.5
**Iron (mg/kg)**^**a**^	32.3	32.8	33.7	35.6	62.4	53.8	177.0
** Heme iron**^**d**^	3.41	3.86	4.53	6.11	27.51	20.68	0.0
** Non-heme iron**^**d**^	28.85	28.96	29.13	29.53	34.88	33.17	177.0

^a^Analyses performed by Eurofins Food & Agro Testing AS (Moss, Norway): calorific value (EN14918/15400/ISO1928, EN 15400:2011, EN 14918:2010, EN14918:2010), fat (NMKL 131, 1989), carbohydrate (total carbohydrates as glucose, Eurofins in-house method based on Luff Schoorl titration), fiber (ISO 5498), protein (NMKL 6), vitamin D3 (EN 12821: 2009–08), total iron (NMKL No 161).

^b^SFA–saturated fatty acids, MUFA–monounsaturated fatty acids.

^c^calculated values; based on reference values from www.matvaretabellen.no.

^d^calculated values; based on the estimation that heme iron accounts for 80% of the total iron in muscle foods.

^e^Diet composition declared by producer.

### Diet production

The experimental diets were produced at the pilot facility of Animalia (Oslo, Norway). Meat and fish were provided by at least two different Norwegian producers. Raw materials were first processed in a bowl cutter machine, and fat and water content of salmon was analyzed using LF-NMR (low field Nuclear Magnetic Resonance) as previously described [27], whereas meat was analyzed with a FoodScan™ Meat Analyzer (Foss, Denmark). Chicken-, pork- and beef fat were then added to the respective meat type to match the fat content of salmon. Safflower seed oil (S2821, Sigma Aldrich) was blended to one batch of beef meat for the preparation of the Beef n-6 diet. All samples were heated Sous Vide in air-tight bags at 70°C (1.5 cm thickness). After 50 minutes, bags were cooled in cold water and their contents mixed with the powder diet. The finished diets were portioned and vacuumed in small plastic bags, providing sufficient food for one cage of 4 to 5 mice for 24h. Diets were stored at -80°C to prevent lipid peroxidation. For diet characterization, samples of all diets were sent to Eurofins Food & Agro Testing AS (Moss, Norway) (Table 1 and S1 Table).

### Intestinal preparations and scoring of intestinal lesions

A/J Min/+ mice were sacrificed at 13 weeks, by cervical dislocation, before colonic and small intestinal preparations were made as previously described [26]. The formalin-fixed, methylene blue stained preparations were examined by transillumination in an inverted microscope. Intestinal lesions were measured with an eyepiece reticle and the location of each lesions was registered in intervals of 1 cm along the length of the intestine. Based on this data, the number of lesions, average size of lesions and load of lesions (total areal covered by lesions) were assessed for each individual animal. In contrast to the brownish-green coloration of healthy epithelium, stained lesions appear bright blue-green and are characterized by having enlarged crypts and compressed luminal openings, which give each lesion a gyrus-like appearance. In the early stages, colonic lesions are defined as flat aberrant crypt foci (flat ACF). Flat ACF usually lay flat against their surrounding epithelium, however, a small number of lesions may appear somewhat polypoid. As these early lesions continuously develop into tumors, the topology of the lesions typically becomes elevated [23,28]. A cut-off point was set, and lesions of ≥0.196 mm^2^ were defined as tumors. Small intestinal tumors have the same physical features as colonic tumors, but are located enclosed within adjacent villi.

### Fecal water preparation and fecal parameters

Fresh feces from mice of each group were pooled, and 1.0 ml distilled water were added to 400 mg feces. Fecal water was prepared as previously described [25]. TBARS were quantified in fecal water as previously described by Ohkawah et al. [29] and Pierre and colleagues [30], and results are expressed as MDA (malondialdehyde) equivalents. The content of heme was analyzed in fecal water by fluorescence as described by Van Den Berg et al. [31] and Pierre and colleagues [25]. For the analysis of fecal water cytotoxicity (MTT assay), fecal water was diluted 1:20 with culture medium and added to cultivated *Apc*^-/+^ cells derived from C57BL/6J Min/+ mice [32]. The MTT assay, as well as the validation and authentication of Apc^-/+^ cells was performed as previously described [33,34].

### Statistics and data presentation

All tests were conducted using 5% significance level. Colonic tumor incidence, meaning the rate of colonic tumor formation, was low in the muscle food groups, and dependency between diet and the colonic tumor incidence was compared by chi-square independency test. One-way ANOVA testing on log-transformed responses for the total number, average size and load (total area covered) of colonic flat ACF and small intestinal tumors was used to identify differences between experimental groups, applying the model:

$$y_{ji}=\mu +\tau_{j}+\epsilon_{ji},\;\sum \tau_{j}=0,\;\epsilon_{i}∼N\left(0,\sigma^{2}\right),\;i=1,\ldots ,n_{j}\;j=B,\ldots ,RM1$$ (1)

where y_ji_ denotes the response variable, μ denotes the overall mean for all groups and τ_j_ the effects of treatment j (7 in total, abbreviations used as subscripts). Model assumptions were controlled by visual inspection of the Q-Q plot of residuals against theoretical normal quantiles, and residuals vs. predicted values. For responses with significant overall effect post hoc multiple comparison analyses were conducted by testing contrasts, defined prior to the analysis, using Scheffé’s method, which accounts for the multiple testing within each response variable. To assess the adequacy of the number of animals included in the study, minimum effect sizes were calculated for the load of intestinal lesions that were required to reveal significant differences between two sample means. Calculations were based on the observed standard deviations of the parameters (log-transformed), various levels of power (20%, 50% and 80%), and the significance level of 5%. Body weight and food intake were analyzed by two-way ANOVA, using diet and gender as independent factors. Box plots indicate the median, and the 10^th^, 25^th^, 75^th^ and 90^th^ percentiles as vertical boxes with error bars, and dots indicate data points. For the presentation of the tumor load distribution along the intestine, intestinal lesions were allocated into 5 location categories, encompassing the area of 0–19%, 20–39%, 40–59%, 60–79% and 80–100% of the small intestine and colon (proximal to distal).

Principal component analysis (PCA) on experimental groups was performed using parameters of intestinal carcinogenesis and fecal parameters, and total amounts of individual constituents of the experimental diets as variables. To adjust the large differences in magnitudes of variables, weighing by 1/Sdev was used.

## Results

### Animal body weight, diet consumption and stability of experimental diets

There were no significant differences in initial body weight between genders or study groups at weaning at three weeks (p = 0.79), whereas final weight was differentially affected by gender and diet (p<0.01). At the end of the study, male animals weighted more than female animals (p<0.01) (Table 2), and animals on meat and fish diets had a significantly greater final body weight than animals in the RM1 group (p<0.01). Yet, average energy intake during the final week of the study did not differ between groups or gender (p = 0.59) (Table 2). TBARS levels were analyzed in the fresh diets and diet leftovers after 24 h in the cage (S1 Fig). In fresh diets, TBARS levels were generally low, with the highest concentration analyzed in Salmon. The highest rates of TBARS formation after 24h were found for Salmon and Beef n-6, while the remaining diets proved to be relatively unaffected by peroxidation.

**Table 2:** Size of study groups, body weight and daily energy intake.

	Powder-based muscle food diets						Reference diet
	Salmon	Chicken low fat	Chicken	Pork	Beef	Beef n-6	RM1
**N (male/female)**	9/9	9/9	9/9	9/10	10/9	9/9	9/9
**Final body weight, total (g)**	22.8 [21.1–24.5]	22.8 [21.4–24.2]	22.2 [20.5–24.0]	23.0 [21.6–24.3]	22.6 [21.5–23.8]	23.0 [21.5–24.5]	19.9 [19.2–20.5]
**Final body weight, male (g)**	25.3 [24.0–26.7]	25.1 [23.8–26.5]	25.6 [24.3–26.9]	24.6 [23.3–25.9]	24.0 [22.7–25.3]	25.5 [24.2–26.9]	21.1 [19.8–22.5]
**Final body weight, female (g)**	20.3 [19.0–21.7]	20.5 [19.1–21.8]	18.9 [17.5–20.2]	21.5 [20.2–22.8]	21.2 [19.8–22.5]	20.4 [19.1–21.8]	19.0 [17.6–20.3]
Energy intake (KJ/animal*day)	45 [43–47]	49 [44–53]	45 [40–50]	45 [42–49]	45 [41–48]	46 [43–50]	48 [45–52]

Results are presented as mean [95% confidence interval].

### Effects of muscle foods on intestinal carcinogenesis

**Fig 1:**
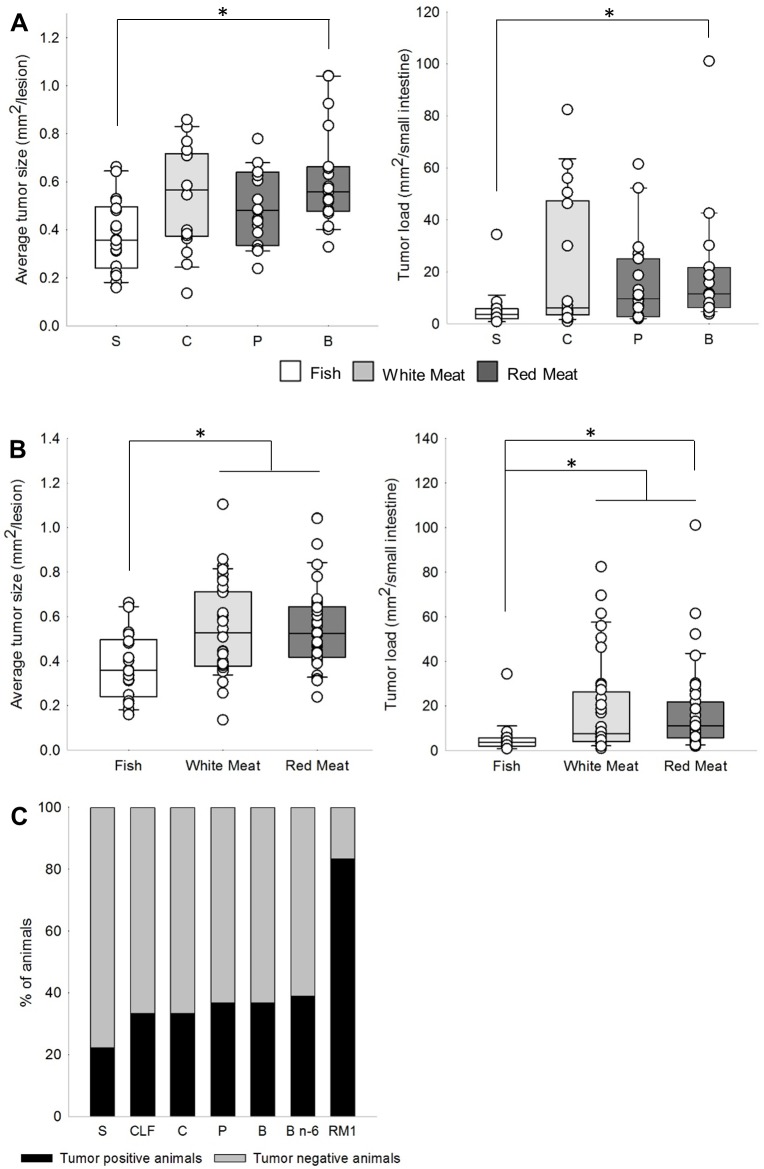
Effects of experimental diets on intestinal carcinogenesis. (A) Effects of Salmon [S], Chicken [C], Pork [P] and Beef [B] on average small intestinal tumor size and tumor load. (B) Effects of Fish [Salmon], white meat [Chicken, Chicken Low Fat] and red meat [Pork, Beef, Beef n-6] on average small intestinal tumor size and tumor load. (C) Effect of Salmon [S], Chicken Low Fat [CLF], Chicken [C], Pork [P], Beef [B], Beef n-6 [B n-6] and RM1 on colonic tumor incidences [proportion of tumor positive and negative animals]. Significant differences are indicated by asterisks.

Results from one-way ANOVA showed that the experimental diets had significantly different effects on the number and load of colonic flat ACF, and on the number, average size and load of small intestinal tumors (Table 3). Contrast analyses revealed that Beef, Pork, Chicken and Salmon with similar fat content did not differentially affect the number, average size or load of flat ACF in the colon. In the small intestine, the number of tumors did not significantly differ between the four groups (Table 4, S2 Fig); however, when compared to Salmon, Beef resulted in a significantly increased average tumor size (p = 0.01) and tumor load (p = 0.03) in the small intestine (Fig 1A).

**Table 3:** Results from one-way ANOVA of the effects of the experimental diets on intestinal carcinogenesis.

	Colonic flat ACF			Small intestinal tumors		
	number	average size	load	number	average size	load
(Intercept)	2.82	-5.05	-2.23	2.95	-0.72	2.23
Beef	-0.09	-0.07	-0.17	0.13	0.18	0.32
Beef n-6	-0.21	-0.06	-0.27	-0.15	-0.06	-0.21
Pork	0.14	0.02	0.17	-0.01	-0.03	-0.04
Chicken	-0.26	-0.03	-0.23	-0.04	0.00	-0.03
Chicken Low Fat	-0.72	-0.06	-0.79	-0.05	0.07	0.02
Salmon	-0.63	-0.06	-0.69	-0.70	-0.32	-1.02
RM1	1.78	0.20	1.98	0.81	0.16	0.97
sigma_sq (σ^2^)	1.45	0.32	1.91	0.65	0.14	1.13
F-value	8.78	0.55	8.17	5.47	3.85	5.66
p-value	**<0.01**	0.77	**<0.01**	**<0.01**	**<0.01**	**<0.01**

Columns indicate the 6 different responses. Rows 1–8 show estimates for the main effect parameters (τ), row 9 gives the error variance estimates (mean square error), and row 10 and 11 gives the F-statistics (at df 6 and 121) and p-values from one-way ANOVA. Significant results (p<0.05) are shown in bold text.

**Table 4:** Results from Scheffé’s method for multiple testing of the log-transformed number, average size and load of colonic flat ACF and small intestinal tumors (post hoc).

			Colonic flat ACF			Small intestinal tumors		
Test	Contrast		number	average size	load	number	average size	load
Effects of diets with similar fat content	Beef—Pork *τ*_*B*_ − *τ*_*P*_	*Estimate*	-0.23	-0.10	-0.33	0.14	0.22	0.36
		p-value	1.00	1.00	1.00	1.00	0.77	0.98
	Beef—Chicken *τ*_*B*_ − *τ*_*C*_	*Estimate*	0.17	-0.11	0.07	0.17	0.18	0.35
		p-value	1.00	1.00	1.00	1.00	0.9	0.98
	Beef—Salmon *τ*_*B*_ − *τ*_*S*_	*Estimate*	0.54	-0.01	0.53	0.83	**0.51**	**1.34**
		p-value	0.93	1.00	0.97	0.14	**0.01**	**0.03**
	Pork—Chicken *τ*_*P*_ − *τ*_*C*_	*Estimate*	0.41	-0.01	0.40	0.03	-0.04	0.00
		p-value	0.98	1.00	0.99	1.00	1.00	1.00
	Pork—Salmon *τ*_*P*_ − *τ*_*S*_	*Estimate*	0.78	0.08	0.86	0.69	0.29	0.98
		p-value	0.70	1.00	0.73	0.35	0.46	0.25
	Chicken—Salmon *τ*_*C*_ − *τ*_*S*_	*Estimate*	0.37	0.09	0.46	0.66	0.33	0.99
		p-value	0.99	1.00	0.99	0.42	0.33	0.26
Effects of fat	Chicken—Chicken Low Fat *τ*_*C*_ − *τ*_*CLF*_	*Estimate*	0.46	0.09	0.55	0.01	-0.06	-0.06
		p-value	0.97	1.00	0.96	1.00	1.00	1.00
	Beef—Beef n-6 *τ*_*B*_ − *τ*_*B n*−6_	*Estimate*	0.12	-0.02	0.10	0.29	0.24	0.53
		p-value	1.00	1.00	1.00	0.98	0.69	0.89
Effects of food groups	Red Meat—White Meat $\frac{1}{3}(\tau_{B}+\tau_{B\;n-6}+\tau_{P})-\frac{1}{2}(\tau_{C}+\tau_{CLF})$	*Estimate*	0.49	-0.01	0.48	0.25	0.12	0.37
		p-value	0.61	1.00	0.77	0.85	0.84	0.77
	Red Meat—Fish $\frac{1}{3}(\tau_{B}+\tau_{B\;n-6}+\tau_{P})-\tau_{S}$	*Estimate*	0.58	0.02	0.60	0.69	**0.36**	**1.04**
		p-value	0.79	1.00	0.86	0.14	**0.06**	**0.05**
	White Meat—Fish $\frac{1}{2}(\tau_{C}+\tau_{CLF})-\tau_{S}$	*Estimate*	0.14	0.04	0.18	0.66	0.36	1.02
		p-value	1.00	1.00	1.00	0.25	0.09	0.10
	Meat—Fish $\frac{1}{5}(\tau_{B}+\tau_{B\;n-6}+\tau_{P}+\tau_{C}+\tau_{CLF})-\tau_{S}$	*Estimate*	0.40	0.03	0.43	0.68	**0.36**	**1.03**
		p-value	0.94	1.00	0.96	0.11	**0.04**	**0.03**
Effects of basic diets	RM1 vs. Powder-based diets $\tau_{RM1}-\frac{1}{6}(\tau_{B}+\tau_{B\;n-6}+\tau_{P}+\tau_{C}+\tau_{CLF}+\tau_{S})$	*Estimate*	**2.08**	0.24	**2.31**	**0.94**	0.19	**1.13**
		p-value	**0.00**	0.85	**0.00**	**0.00**	0.69	**0.01**

Each part consist of two rows showing the estimate for the contrast as defined in column 2, and the p-value for the hypothesis that the true contrast is zero. The τ‘s are the main effects of the experimental diets, and refer directly to the model defined by Eq (1). The subscripts indicate the experimental diets, with Salmon [S], Chicken Low Fat [CLF], Chicken [C], Pork [P], Beef [B], Beef n-6 [B n-6] and RM1 [RM1]. Significant results (p<0.05) are shown in bold text.

Results from contrast analyses, investigating the effects of fat on colonic and small intestinal carcinogenesis, showed that there was no significant difference between the effects of the two chicken diets, where fat levels, but not fatty acid composition differed. Likewise, a higher concentration of n-6 unsaturated fat in beef meat did not significantly affect the development of colonic flat ACF or small intestinal tumors, as there was no difference between mice fed Beef and Beef n-6 (Table 4 and S2 Fig).

For further analyses, the muscle food groups were grouped into Red Meat (Beef, Beef n-6, Pork), White Meat (Chicken, Chicken Low Fat) and Fish (Salmon). Chicken Low Fat was included into the White Meat group and Beef n-6 was included into the Red Meat group, based on the findings that the formation of intestinal lesions was not affected by dietary fat. Again, no differences were found for colonic carcinogenesis. Also, no significant differences were found when the effects of Red Meat were compared to White Meat in the small intestine. However, Red Meat induced a greater small intestinal tumor load (p = 0.05) than Fish, and the difference was reflected by a borderline significant increase in average tumor size (p = 0.06) (Table 4 and Fig 1B). Indications are also given that White Meat resulted in an increased average small intestinal tumor size (p = 0.09) and tumor load (p = 0.10), when compared to Fish. Accordingly, Red Meat and White Meat combined induced a significantly greater average tumor size (p = 0.04) and tumor load (p = 0.03) than Fish.

**Fig 2:**
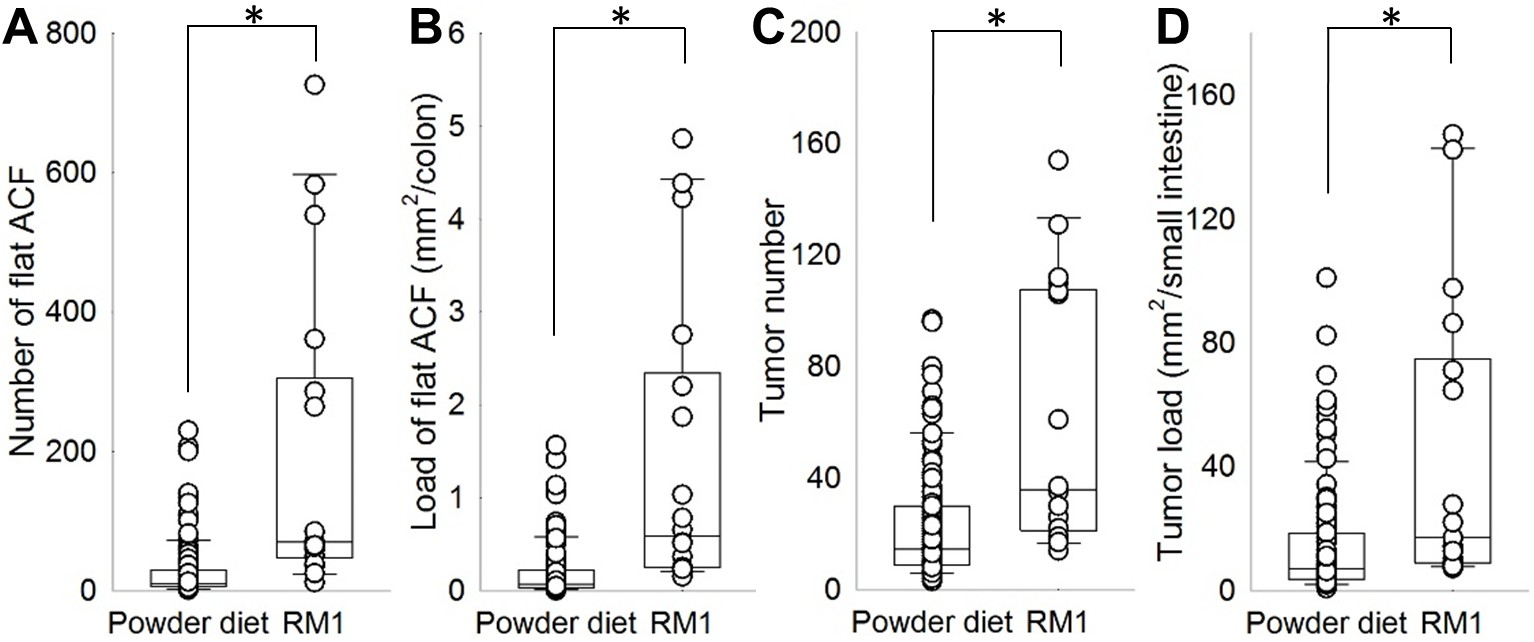
Effects of the powder diet (all powder-based muscle food diets combined) and RM1 on number and load of intestinal lesions. (A) Number of colonic flat ACF, (B) Load of colonic flat ACF, (C) Number of small intestinal tumors, (D) Load of small intestinal tumors. Significant differences are indicated by asterisks.

The reference diet RM1 differed fundamentally in its composition from the muscle food diets based on the standardized powder diet. When comparing the effects of RM1 to the muscle food diets in order to examine the effects of the basic diets on intestinal carcinogenesis, mice fed RM1 showed a significantly larger number and load of colonic flat ACF (both p<0.01) and small intestinal tumors (p<0.01 and p = 0.01, respectively) (Table 4 and Fig 2). The average size of lesions was not affected (S2 Fig). In regard to tumor formation in the colon, tumor incidence varied significantly between the experimental groups (p<0.01, χ^2^ = 17.2 at df 6). Fig 1C indicates a larger probability of tumor development in mice fed RM1 and a slight protective effect of salmon on tumor incidence.

**Fig 3:**
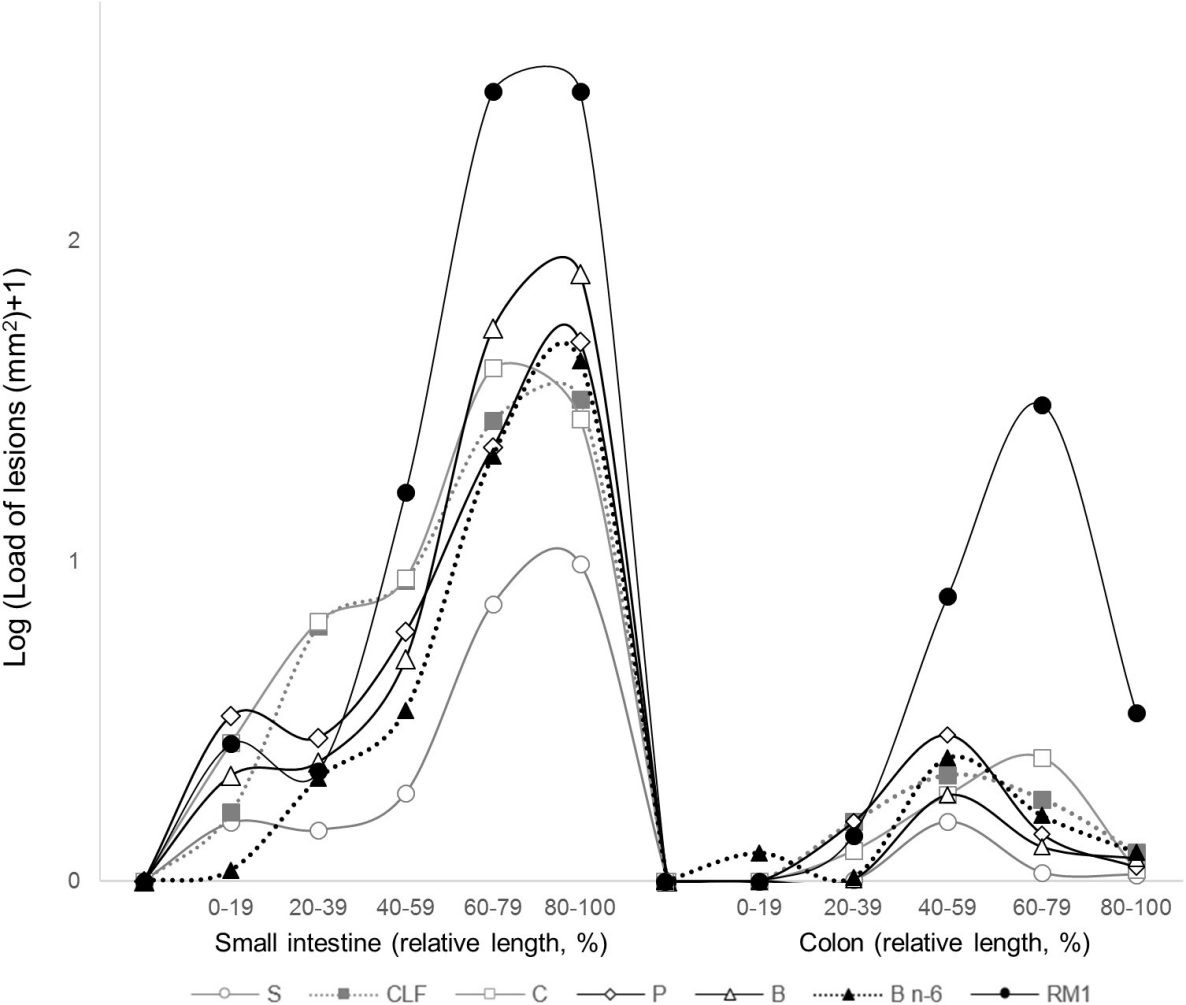
Effects of experimental diets on the load of lesions along the relative length of the intestine. Salmon [S], Chicken Low Fat [CLF], Chicken [C], Pork [P], Beef [B], Beef n-6 [B n-6]. Values represent means from log-transformed data.

Fig 3 presents the average area covered by lesions (load) along the intestine. All study groups present a similar distribution, where the greatest load of intestinal lesions is found in the distal sections of both the small intestine and the colon. The graph shows that mice fed the reference diet RM1 had a greater tumor load than mice fed any of the other diets, in both small intestine and colon, and that differences between the powder-based experimental groups were comparatively modest. Only the tumor load profile of mice fed Salmon appeared to be lower than the profiles of mice fed meat diets, and the difference was more pronounced in the small intestine than in the colon. Size distributions of intestinal lesions (S3 Fig) further illustrate the presented results.

To test the robustness of our results, gender and parents were separately added as additional predictors. Importantly, the number of animals versus variables reduces the degrees of freedom considerably, especially for “parents”. The effects of gender were in general small, whereas the effects of parents (26 combinations of dam and sire) were clear for most responses, indicating genetic or environmental factors for the interindividual variation. The effects of parents on the estimates for the significant one-way contrasts, however, were small, but led to smaller differences, i.e. marginally larger p-values.

To evaluate whether the group size of 20 animals was adequate for the detection of relevant differences between groups in the present study, minimum detectable effect sizes were determined for the load of flat ACF, colonic tumors and small intestinal tumors (Table 5). The calculations were based on the observed standard deviations of the log-transformed means, and revealed that the size of the effects required to detect significant differences at a significance level of 5% were within expectable ranges. However, real minimum detectable effects of the tested contrasts in the present study (Table 4) must be assumed to be slightly larger, as contrast analyses (Scheffé’s method), as opposed to power calculations, accounted for multiple testing.

**Table 5:** Minimum detectable effect sizes for testing whether two means are different under the given circumstances of the present study (load of flat ACF, load of colonic tumors and load of small intestinal tumors (log-transformed)).

Parameter	N	Standard deviation	Power	Minimum detectable effect	Reference values		
					Salmon	Beef	RM1
Load of colonic flat ACF	18	1.38	0.8	**1.33**	-2.92	-2.39	-0.25
			0.5	**0.93**			
			0.2	**0.53**			
Colonic tumor load		0.86	0.8	**0.82**	0.16	0.28	1.66
			0.5	**0.58**			
			0.2	**0.33**			
Small intestinal tumor load		1.06	0.8	**1.02**	1.21	2.55	3.20
			0.5	**0.71**			
			0.2	**0.41**			

Calculations were based on the observed standard deviations of the log-transformed means, and a group size of 20 animals. Three different levels of power were chosen, and calculated values for the minimum detectable effects are given. Exemplary log-transformed reference values for the parameters of the Salmon, Beef and RM1 group illustrate the relevance of the effect sizes.

### Fecal water analyses

**Fig 4:**
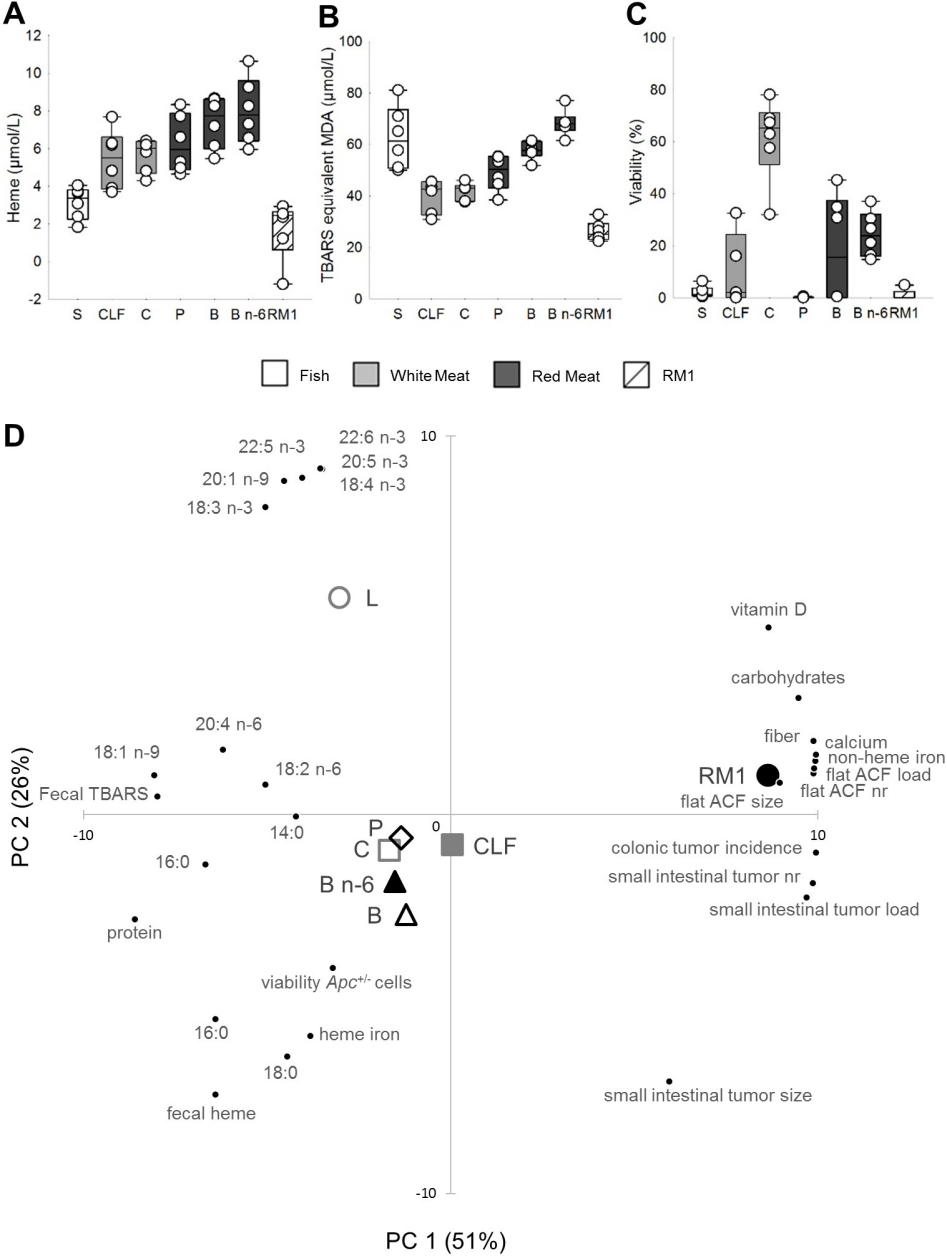
Fecal water analyses and scatter plot of PCA on experimental groups. Fecal water content of (A) heme and (B) TBARS, and (C) cytotoxic effect of fecal water on *Apc*^-/+^ cells (n = 1, fecal water from pooled fresh feces of 18 to 19 animals per group). Data points and box plots indicate measurement uncertainties of the methods. (D) PCA biplot showing scores from PC1 and PC2: associations between experimental groups, and parameters of intestinal carcinogenesis and fecal water, and constituents of the experimental diets. Salmon [S], Chicken Low Fat [CLF], Chicken [C], Pork [P], Beef [B], Beef n-6 [B n-6].

Fecal heme concentrations (Fig 4A) were highest in the sample from animals fed Beef n-6, followed by Beef, Pork, Chicken, Chicken Low Fat and Salmon. The sample from mice fed RM1 presented the lowest concentrations of fecal heme. The highest concentration of fecal TBARS (Fig 4B) were found in the sample from animals fed Salmon and Beef n-6, followed by samples from animals fed Beef, Pork and the Chicken diets. Fecal TBARS concentration were lowest in the sample from the RM1 group.

*Apc*^-/+^ cells were incubated with fecal water to assess fecal water cytotoxicity (Fig 4C). Fecal water samples from mice fed Salmon, Pork and RM1 induced the greatest reduction of cell viability, while the highest cell viability of all experimental groups was observed in response to fecal water of mice fed Chicken.

### Principal component analysis (PCA)

PCA (Fig 4D) shows the relationship between characteristics of fecal water, constituents of the experimental diets and intestinal carcinogenesis. PC 1 and 2 explained more than 75% of variance. Experimental groups fed meat from terrestrial animals clustered around the intersection of PC 1 and 2, while the group fed Salmon was better defined by PC 2, i.e. high levels of unsaturated fatty acids in the diet and low heme content. The RM1-group was associated with parameters of intestinal carcinogenesis, dietary non-heme iron, calcium, vitamin D, carbohydrates and fiber. Colonic tumor development was negatively correlated to fecal heme and TBARS.

## Discussion

In epidemiological and experimental studies, high intake of red meat has been associated with an increased risk for CRC. Fish consumption may have a protective effect on CRC, while meat from poultry is considered neutral [7]. To our knowledge, this is the first experimental study to directly compare the effects of intake of red meat (beef and pork), white meat (chicken) and fish (salmon) with similar fat content on intestinal carcinogenesis. Results indicate that replacing beef with salmon may reduce small intestinal tumor burden in A/J Min/+ mice, and more generally, fish intake, represented by salmon, was found to result in lower cancer burden than meat from terrestrial animals (approx. factor 3). Similarly, a protective effect of dietary long chain n-3 PUFA from fish oil on intestinal carcinogenesis has previously been documented in the conventional C57BL/6 Min/+ mouse [35,36]. In the present study, white meat did not prompt a more beneficial outcome than red meat, but instead affected intestinal carcinogenesis in the same manner as red meat. Also, tumor burden induced by the chicken diet (16.8% fat) did not differ from the tumor burden in mice fed the low-fat chicken diet, a diet characterized by a similar composition of fatty acids but lower levels of fat (4.5%). This gives reason to assume that the lack of differences between the effects of red and white meat was not attributed to the relatively high level of fat in the chicken diet. One of the main hypothesis linking red meat to an increased risk of CRC, concerns the idea that heme iron from red meat enhances oxidative stress through lipid peroxidation [5,6,25]. Thus, the combination of heme iron and a high level of PUFA may adversely affect intestinal health [17,18]. However, the comparison of the effects of dietary beef with the effects of dietary beef with additional n-6 PUFA indicated that the effects of beef on small intestinal and colonic carcinogenesis were not modulated by the quality of fat in the present study. The results of the present study do not give any indications of a role of heme iron in CRC.

Remarkably, the largest tumor burden, in both the small intestine and colon was induced by the reference diet RM1. Albeit similar tumor initiating effects of RM1 have been reported before [26,37], the result is surprising due to various reasons: In contrast to the semi-synthetic powder diet, the natural ingredient diet RM1 was not adjusted for nutrients with documented protective properties, e.g. calcium, vitamin D or fiber. Moreover, despite the similar calorie intake between study groups, consumption of RM1 resulted in a lower body weight, which is considered beneficial in regard to CRC risk [8]. Diet compositions of the powder-based diets differ from RM1 in many aspects, which makes it impossible to assess whether the observed differences between the basic diets (RM1 and powder diet) may be attributed to the level of particular macronutrients, micronutrients or unknown, bioactive compounds. Nevertheless, based on the PCA biplot, it may be speculated that the tumor inducing potential of the RM1 feed is, to some extent, connected to the high level of organic iron, which was previously shown to enhance intestinal carcinogenesis in Min/+ mice [38]. Besides, the outcome may be associated with the higher proportion of energy provided by carbohydrates [8]. Most importantly, the results confirm the model’s responsiveness to dietary stimuli, and underline that differences provoked by the different muscle foods were small in comparison to differences provoked by unknown factors in the basic diets. Nutritional factors that are typically included as confounding variables in epidemiological CRC risk assessments are total energy intake, fiber, calcium, folate and use of multivitamin supplements [1,2,39]. As meat and fish meals may be typically consumed along with certain accompaniments (e.g. vegetables, potatoes or bread) [40], the results emphasize the importance of the inclusion of certain food groups and macronutrients as putative confounding factors in epidemiological studies.

Several experimental studies link lipid peroxidation and fecal water cytotoxicity to intestinal carcinogenesis [25,41]. Fecal TBARS formation appeared to be enhanced by unsaturated fat and heme iron, but in line with the findings from a previous study on A/J Min/+ mice [42], the present study does not indicate a direct link between luminal peroxidation and intestinal carcinogenesis. Moreover, fecal water cytotoxicity on *Apc*^-/+^ cells of the various experimental groups did not follow an evident pattern, and there was no indication for a relationship between fecal water cytotoxicity on *Apc*^-/+^ cells, fecal TBARS, and intestinal carcinogenesis. Despite the favorable effect of dietary salmon on small intestinal carcinogenesis, levels of TBARS and fecal water cytotoxicity were amongst the highest in response to salmon. In turn, a strong fecal water cytotoxicity in the RM1 groups coincided with high rates of tumor formation, but was not connected to lipid peroxidation. Hence, the use of TBARS in predicting carcinogenic effects, and the relevance of the cytotoxicity assay in the present study seem controversial. The MTT assay that was used to assess fecal water cytotoxicity, does only indicate decreases in cell viability, and does not allow to differentiate between apoptotic and necrotic processes. Hence, it needs to be established whether the induction of cell death of epithelial cells presents an advantage or disadvantage in regard to intestinal carcinogenesis.

Ingested heme iron is presumed to increase fecal water cytotoxicity, and heme in the soluble fraction of the feces is thought to interact more strongly with the intestinal epithelium than the non-soluble fraction [33]. Despite of higher fecal concentrations of heme in response to dietary red meat than white meat and fish, concentrations were low in comparison to fecal concentrations detected in rats fed equivalent amounts of beef or heme iron [30,33,34,43]. The amount of heme iron reaching the colon may be influenced by possible precipitation of heme by compounds like calcium [30,44], but calcium levels in the muscle food diets were low (0.06–0.07%). Besides, fecal heme concentration is determined by the absorption rate of heme in the small intestine. As opposed to humans, in rats and mice, heme iron is absorbed at lower rates than non-heme iron [45,46], and the adaption of the absorption of heme is limited even in case of iron deficiency [47]. Nevertheless, absorption rates of heme and non-heme iron were shown to correlate in rats [45], and in comparison with C57BL/6J wild type mice, mice on an A/J genetic background were shown to absorb free iron twice as efficient [48]. Hence, the discrepancies in the effects of red meat between the present study and previously performed studies by Pierre and colleagues [30,33] may be related to a more efficient removal of intestinal heme in A/J Min/+ mice. More work is necessary to evaluate the translation potential of rodent studies in regard to heme metabolism.

Carcinogenesis is divided into initiation, promotion and progression, and, depending on their mode of actions, carcinogens have the ability to interfere with molecular processes at either stage of tumor development [49]. The period where the majority of new intestinal lesions are spontaneously initiated in A/J Min/+ mice covers the time span from birth to approximately 30 weeks of age, and peaks at the age of 7 to 12 weeks. While small intestinal lesions are characterized by a relatively uniform growth throughout the lifespan of the mouse, an extensive growth acceleration in the colon is not seen before the age of 30 weeks [23]. In the present work, similar tendencies of tumor induction were observed in the small intestine and colon after 13 weeks, but differences between study groups were more pronounced in the small intestine, where the average size and load of tumors was significantly increased in response to meat from terrestrial animals. Thus, it needs to be established how intestinal lesions, and, in particular, colonic lesions, are influenced by different muscle foods during later stages of tumor development. Nevertheless, calculations of the minimum effect sizes required to detect significant differences between groups at this early stage of intestinal carcinogenesis indicated that a sufficient number of animals was included in the present study.

The possibility that CRC may not be influenced by red meat at the stage of tumor initiation is supported by a long-term study conducted by Winter et al. [50], where no increased rate of colonic neoplasms was observed in heme iron-fed C57BL/6 WT mice, and two previous studies on A/J Min/+ mice that documented an inhibitory effect of heme iron on colonic carcinogenesis [26,42]. In rodent studies that reported promoting effects of red meat, intestinal carcinogenesis was accelerated by azoxymethane (AOM) or 1,2-demethylhydrazine (DMH) [30,33,43]; two colon-specific carcinogens, which stimulate the acquisition of mutations in key regulatory genes.

Due to an evolutionary loss of the cytidine monophospho-N-acetylneuraminic acid hydroxylase (CMAH), humans, as opposed to other mammals, are not able to endogenously produce N-glycolylneuraminic acid (Neu5Gc) through enzymatic conversion of N-actetylneuraminic acid (Neu5Ac) [51]. Nevertheless, Neu5Gc from dietary sources, e.g. red meat, seems to be incorporated into human tissue and recently, inflammatory processes induced through recognition of Neu5Gc by auto-reactive antigens have been proposed as a mechanism in red-meat related CRC [52]. If this mechanism is proven to be valid, mice with a functional *Cmah* gene, like the A/J Min/+ mouse, will most likely not represent a suitable model for red meat-related CRC. However, crossing A/J Min/+ with *Cmah*^-/-^ mice (mice that do not express Neu5Gc due to deletion of exon 6 of the *Cmah* gene [53]) could provide a helpful tool in the investigation of the role of Neu5Gc in red meat related CRC development.

In summary, the results of the present work do not indicate that the intake of cooked red meat is less favorable for CRC development than the intake of white meat. However, it was shown that consumption of salmon may inhibit intestinal carcinogenesis. The present study could not confirm a link between TBARS, fecal water cytotoxicity and intestinal carcinogenesis, but underlines the importance of the basic diet during carcinogenesis. Long-term studies are needed to increase knowledge on the effects of red meat on initiation, promotion and progression of CRC in A/J Min/+ mice.

## Supporting information

S1 Table Fatty acid composition of the experimental diets. (PDF)

S2 Table Ingredients of the powder diet and RM1. (PDF)

S3 Table Composition of the powder diet. (PDF)

S1 Fig TBARS concentrations in fresh muscle food diets (T0) and after 24h in the cage (T24). Salmon [S], Chicken Low Fat [CLF], Chicken [C], Pork [P], Beef [B] and Beef n-6 [B n-6].
(PDF)

S2 Fig Effect of Salmon (S), Chicken Low Fat (CLF), Chicken (C), Pork (P), Beef (B), Beef n-6 (B n-6) and RM1 on the number, average size and load of intestinal lesions in A/J Min/+ mice. (PDF)

S3 Fig Size distribution of (A) flat ACF and tumors in the colon, and (B) small intestinal tumors of A/J Min/+ mice fed Salmon [S], Chicken Low Fat [CLF], Chicken [C], Pork [P], Beef [B], Beef n-6 [B n-6] and RM1.
(PDF)

S1 File Raw data. (XLSX)
