# Supplementary material for: Effects of dietary beef, pork, chicken and salmon on intestinal carcinogenesis in A/J Min/+ mice
Source: PLoS One. 2017 Apr 20;12(4):e0176001. doi: 10.1371/journal.pone.0176001 (PMC5398569; doi:10.1371/journal.pone.0176001)
Supplement: S1 Table — (PDF) [file pone.0176001.s001.pdf]

S1 Table: Fatty acid composition of the experimental diets.

	Powder-based muscle food diets						Reference diet
	**Salmon**	**Chicken Low Fat**	**Chicken**	**Pork**	**Beef**	**Beef n-6**	**RM1**
**% of total fatty acids**[a]							
**C14:0**	2.2	1.2	0.8	1.5	3.1	2.2	5.2
**C16:0**	8.8	23.2	21.2	22.0	23.6	19.1	11.4
**$C16:1_{n-7}$**	2.5	4.2	4.2	2.3	2.9	2.1	3.3
**C18:0**	2.4	6.6	6.0	10.5	21.0	16.0	1.5
**$C18:1_{n-9}$**	42.9	39.6	40.1	43.6	36.1	31.4	28.4
**$C18:2_{n-6}$**	13.4	17.6	21.2	12.8	2.5	19.9	25.5
**$C18:3_{n-3}$**	4.8	1.7	2.1	1.4	0.8	0.5	2.2
**$C18:4_{n-3}$**	0.6	-	-	-	-	-	-
**$C20:1_{n-9}$**	3.7	0.4	0.4	0.9	0.2	0.2	-
**$C20:4_{n-6}$**	0.3	0.6	0.4	0.4	0.1	0.1	-
**$C20:5_{n-3}$**	2.6	-	-	-	-	-	-
**$C22:5_{n-3}$**	1.6	0.2	-	0.2	0.1	0.1	-
**$C22:6_{n-3}$**	3.9	0.2	-	0.1	-	-	-
**DB**[b]	**138.3**	**89.1**	**95.0**	**79.8**	**47.5**	**75.9**	**89.3**

[a]Analyses performed by Eurofins Food & Agro Testing AS (Moss, Norway): fatty acid composition [1]

[b]DB is the relative number of double bonds per g fat, and was calculated by the formula:
DB= $\sum_{n=1}^{6} n(\%\text{ fatty acids with n double bonds})$

1. Ottestad I, Vogt G, Retterstøl K, Myhrstad MC, Haugen J-E, Nilsson A, et al. Oxidised fish oil does not influence established markers of oxidative stress in healthy human subjects: a randomised controlled trial. Br J Nutr. 2012 Jul;108(2):315–26.
