# Supplementary material for: Effects of dietary beef, pork, chicken and salmon on intestinal carcinogenesis in A/J Min/+ mice
Source: PLoS One. 2017 Apr 20;12(4):e0176001. doi: 10.1371/journal.pone.0176001 (PMC5398569; doi:10.1371/journal.pone.0176001)
Supplement: S2 Table — (PDF) [file pone.0176001.s002.pdf]

S2 Table: Ingredients of the powder diet and RM1.

Powdered diet	RM1
Rice Starch	Wheat
Sucrose	Barley
Casein	Wheat feed
AIN-93G-MX, adjusted for Ca and P	De-hulled extracted Toasted Soya
Cellulose	Soya Protein Concentrate
AIN-93-VX, without supplementary vitamin D3	Macro Minerals
L-Cystine	Soya Oil
Choline Bitatrate	Whey powder
	Amino Acids
	Vitamins
	Micro Minerals
