# Supplementary material for: Effects of dietary beef, pork, chicken and salmon on intestinal carcinogenesis in A/J Min/+ mice
Source: PLoS One. 2017 Apr 20;12(4):e0176001. doi: 10.1371/journal.pone.0176001 (PMC5398569; doi:10.1371/journal.pone.0176001)
Supplement: S3 Table — (PDF) [file pone.0176001.s003.pdf]

S3 Table: Composition of the powder diet.

	Powder diet
Energy (MJ/kg)	14.7
Fat (g/100 g)	0.0
Protein (g/100 g)	18.3
Sugar as glucose after hydrolysis (g/100 g)	64.6
Fiber (g/100 g)	2.0
adjusted mineral/vitamin level	
total Ca (%)	0.08
total P (%)	0.14
total Vit D3 (iu/100 g)[a]	<18.3

[a]vitamin D from casein (<100 iu/kg casein)
