# Supplementary material for: Effects of dietary beef, pork, chicken and salmon on intestinal carcinogenesis in A/J Min/+ mice
Source: PLoS One. 2017 Apr 20;12(4):e0176001. doi: 10.1371/journal.pone.0176001 (PMC5398569; doi:10.1371/journal.pone.0176001)
Supplement: S1 Fig — Salmon [S], Chicken Low Fat [CLF], Chicken [C], Pork [P], Beef [B] and Beef n-6 [B n-6]. (PDF) [file pone.0176001.s004.pdf]

Stability of the experimental diets

TBARS were analysed in fresh diet and diet leftovers after 24h in the cage. To 2 g of grounded diet, 10 ml of 5% trichloroacetic acid, 40 µl of butylated hydroxytoluene (1 mg/ml) and 40 µl ethylenediaminetetraacetic acid (0.1 M) were added and homogenized for 2 x 15 s with a mechanical grinder. Homogenized mixtures were centrifuged at 3000 x g for 3 minutes at 4°C. The supernatant was collected and filtered through Whatman® qualitative filter paper (grade 1). TBARS were analyzed as previously described by Ohkawah et al. [1].

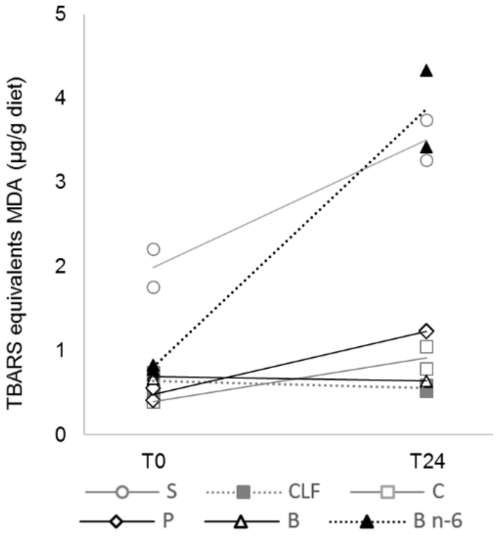

S1 Fig: TBARS concentrations in fresh muscle food diets (T0) and after 24h in the cage (T24). Salmon [S], Chicken Low Fat [CLF], Chicken [C], Pork [P], Beef [B] and Beef n-6 [B n-6].

1. Ohkawa H, Ohishi N, Yagi K. Assay for lipid peroxides in animal tissues by thiobarbituric acid reaction. Anal Biochem. 1979 Jun;95(2):351–8.
