# Supplementary material for: Effects of dietary beef, pork, chicken and salmon on intestinal carcinogenesis in A/J Min/+ mice
Source: PLoS One. 2017 Apr 20;12(4):e0176001. doi: 10.1371/journal.pone.0176001 (PMC5398569; doi:10.1371/journal.pone.0176001)
Supplement: S2 Fig — (PDF) [file pone.0176001.s005.pdf]

Effect of Salmon, Chicken, Pork, Beef and RM1 on intestinal carcinogenesis in A/J Min/+ mice

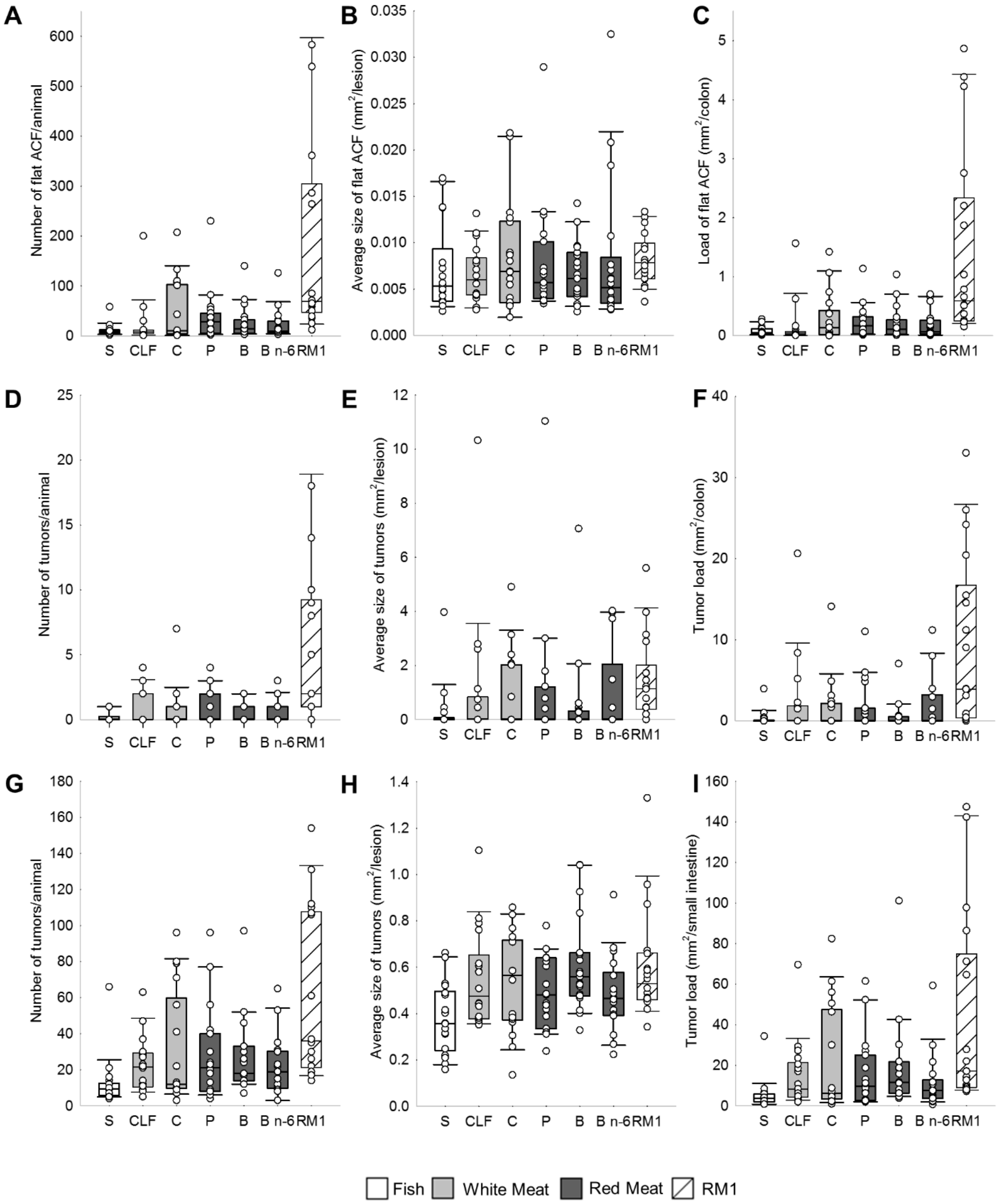

S2 Fig: Effect of Salmon [S], Chicken Low Fat [CLF], Chicken [C], Pork [P], Beef [B], Beef n-6 [B n-6] and RM1 on number, average size and load of intestinal lesions in A/J Min/+ mice. (A-C) flat ACF, (D-F) colonic tumors and (G-I) small intestinal tumors.
