# Supplementary material for: Effects of dietary beef, pork, chicken and salmon on intestinal carcinogenesis in A/J Min/+ mice
Source: PLoS One. 2017 Apr 20;12(4):e0176001. doi: 10.1371/journal.pone.0176001 (PMC5398569; doi:10.1371/journal.pone.0176001)
Supplement: S3 Fig — Size distribution of (A) flat ACF and tumors in the colon, and (B) small intestinal tumors of A/J Min/+ mice fed Salmon [S], Chicken Low Fat [CLF], Chicken [C], Pork [P], Beef [B], Beef n-6 [B n-6] and RM1. (PDF) [file pone.0176001.s006.pdf]

Size distribution of intestinal lesions in A/J Min/+ mice

For the size distribution, intestinal lesions were allocated into five size classes, based on a logarithmic scale: 0-0.008 mm^2; 0.009-0.064 mm^2; 0.065-0.512 mm^2; 0.513-4.096 mm^2 and >4.096 mm^2. Thereby, the smallest lesions (approx. 1-4 lesions) are grouped within the first size class. The presented average numbers of lesions within size classes were calculated from log-transformed data to correct for the large variation between individual mice within the study groups.

Colonic and small intestinal size class distributions (Supplementary Fig 3) illustrate the number of lesions within each size category. It becomes apparent that RM1 had the strongest ability to induce intestinal carcinogenesis in the colon and small intestine of the A/J Min/+ mouse. In the colon, the comparable profiles of the distribution curves illustrated that differences between groups were marginal. In the small intestine, Salmon resulted in a size distribution curve below the other meat diets. In addition, a shift towards larger lesions was only observed for the meat diets from terrestrial animals, but not for Salmon.

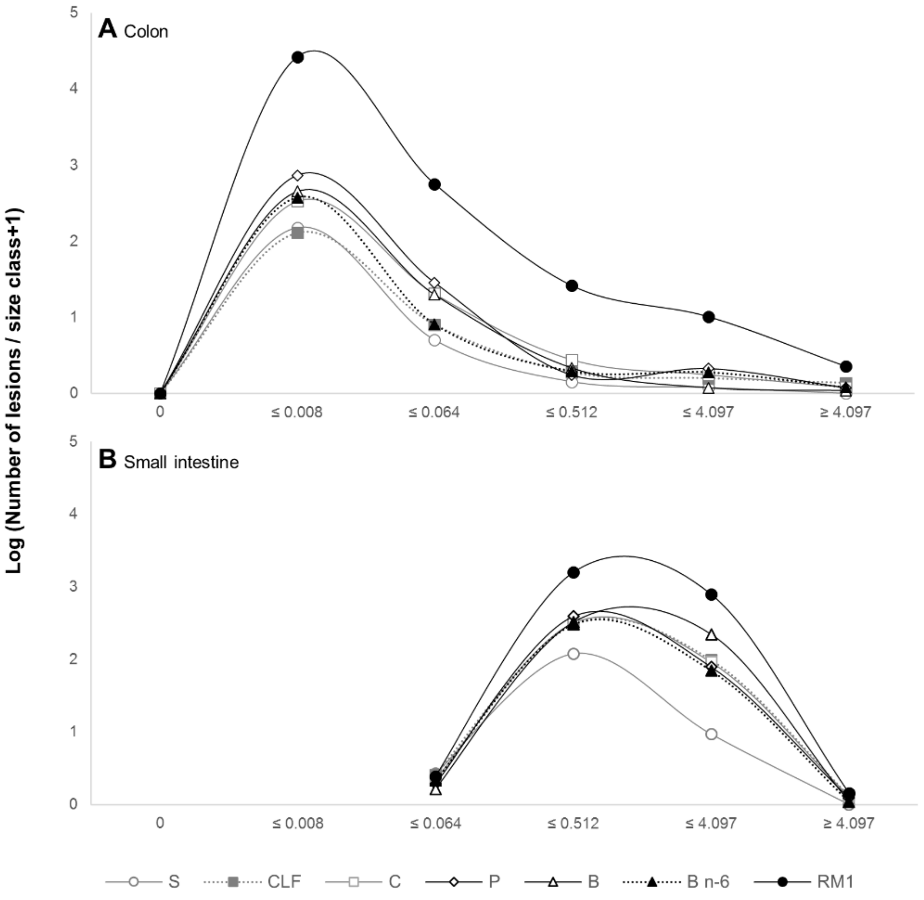

S3 Fig: Size distribution of (A) flat ACF and tumors in the colon, and (B) small intestinal tumors of A/J Min/+ mice fed Salmon [S], Chicken Low Fat [CLF], Chicken [C], Pork [P], Beef [B], Beef n-6 [B n-6] and RM1.
